# A dataset for mobile edge computing network topologies

**DOI:** 10.1016/j.dib.2021.107557

**Published:** 2021-11-08

**Authors:** Bin Xiang, Jocelyne Elias, Fabio Martignon, Elisabetta Di Nitto

**Affiliations:** a Politecnico di Milano, Italy; b University of Bologna, Italy; c University of Bergamo, Italy

**Keywords:** 5G Network, Mobile edge computing, Base stations, Network topology, Geographic location, Random graphs, Network parameters

## Abstract

Mobile Edge Computing (MEC) is vital to support the numerous, future applications that are envisioned in the 5G and beyond mobile networks. Since computation capabilities are available at the edge of the network, applications that need ultra low-latency, high bandwidth and reliability can be deployed more easily. This opens up the possibility of developing smart resource allocation approaches that can exploit the MEC infrastructure in an optimized way and, at the same time, fulfill the requirements of applications. However, up to date, the progress of research in this area is limited by the unavailability of publicly available true MEC topologies that could be used to run extensive experiments and to compare the performance on different solutions concerning planning, scheduling, routing etc. For this reason, we decided to infer and make publicly available several synthetic MEC topologies and scenarios.

Specifically, based on the experience we have gathered with our experiments Xiang et al. [1], we provide data related to 3 randomly generated topologies, with increasing network size (from 25 to 100 nodes). Moreover, we propose a MEC topology generated from OpenCellID [2] real data and concerning the Base Stations’ location of 234 LTE cells owned by a mobile operator (Vodafone) in the center of Milan. We also provide realistic reference parameters (link bandwidth, computation and storage capacity, offered traffic), derived from real services provided by MEC in the deployment of 5G networks.

## Specifications Table

**Table:** 

Subject	Computer Networks and Communications
Specific subject area	Mobile Edge Computing, Edge Networks
Type of data	Network Topologies description in CSV, Table, Graph, Figure
How data were acquired	OpenCellID https://opencellid.org/
Data format	Raw, Analyzed, Filtered
Parameters for data collection	Base stations’ geographic locations for the selected network operators at a specific region
Description of data collection	Extracting data from the OpenCellID website by specifying the geographic region
Data source location	Milan, Italy
Data accessibility	Repository name: GitHub, Zenodo Data identification number: 10.5281/zenodo.5558613 Direct URL to data: https://github.com/bnxng/Topo4MEC
Related research article	B. Xiang, J. Elias, F. Martignon, E. Di Nitto, Resource Calendaring for Mobile Edge Computing: Centralized and Decentralized Optimization Approaches, Computer Networks 199 (2021) 108426. https://doi.org/10.1016/j.comnet.2021.108426

## Value of the Data

• The datasets provide network topologies, computation, storage and traffic parameters that can be used as test instances and benchmarks for evaluating and comparing resource allocation approaches in the context of Mobile Edge Computing. No real network topologies are currently made publicly available in the context of MEC.
• Researchers who work in the field of Mobile Edge Computing can benefit from these datasets.
• The network topologies and parameters provided in this work can be used for performance evaluation in research problems related to MEC like traffic routing, network planning, resource scheduling, among others.

## 1 Data Description

**Fig. 1:**
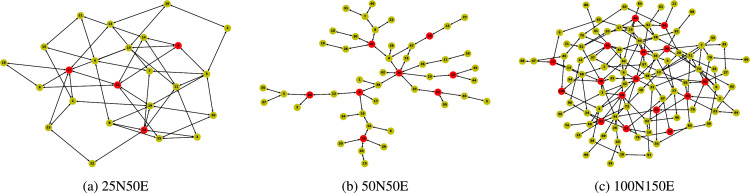
Random network topologies. Ingress nodes for each graph are colored in red. (For interpretation of the references to colour in this figure legend, the reader is referred to the web version of this article.)

**Fig. 2:**
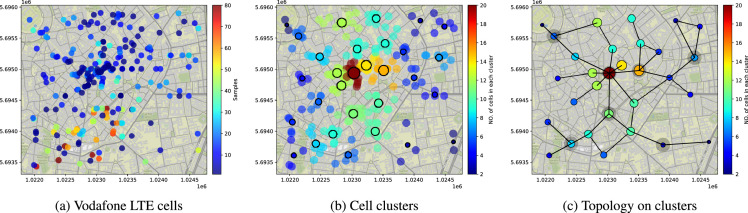
Milan City Center topology with 30 nodes, 35 edges and 6 ingress nodes (marked with gray shadow).

The datasets include four network topologies for Mobile Edge Computing [3] and the corresponding reference parameters for the networks. Both random topologies having different scales (Fig. 1) and a realistic one derived from open datasets for base stations (Fig. 2 c) are provided. The structural information of the topologies is summarized in Table 1, which reports the numbers of nodes, edges, ingress nodes (marked in red in Fig. 1 and with gray shadow in Fig. 2c), as well as the minimum, maximum and average degrees of the nodes, and the network diameter (the longest of all the shortest paths in the network). All topology datasets are publicly available in the GitHub repository. In the repository, the topologies data are provided in four folders, viz., *25N50E, 50N50E, 100N150E* and *MilanCityCenter*. Each folder contains three files: a JPEG image of the topology (also illustrated in Figs. 1 and  2 c hereafter), a text file of the topology data stored in edge-list format (with each row representing one edge/link), and a text file storing the list of the ingress nodes. The topology data (in edge-list format) has three columns, where the first two columns represent, respectively, the first and second nodes of the edge, and the last column represents the bandwidth value of the edge.

**Table 1:** Structural information of the topologies.

Topology name	#Nodes	#Edges	#Ingress	Degree (Min, Max, Avg)	Diameter

*25N50E*	25	50	4	(2.0,7.0,4.0)	4
*50N50E*	50	50	8	(1.0,7.0,2.0)	12
*100N150E*	100	150	16	(1.0,8.0,3.0)	8
*MilanCityCenter*	30	35	7	(1.0,8.0,2.3)	8

Table 2 shows the reference network parameters (as further motivated in Section 2.2) that can be assigned to these network topologies, represented by undirected graphs G(V,E). Each node v∈V represents an *edge computing node* having $D_{v}$ and $S_{v}$ as *computation* and *storage capacity*, respectively. Each *edge* e∈E corresponds to a network link characterized by its *bandwidth* $B_{e}$. Let K denote the set of traffic demands offered to the network from the ingress nodes, which represent aggregated demands of different, typical service classes, e.g., web, video, gaming, etc, and $\lambda^{k}$ is the corresponding arrival rate. Link bandwidth and traffic rates are expressed in Gigabits/s, the storage capacity in Gigabytes while the computation capacity is expressed in Giga cycles/s. Besides the reference value, different ranges are further indicated for all these parameters to capture different configurations and traffic levels.

**Table 2:** Reference values and ranges for network parameters in the considered topologies.

Parameter	Reference value	Range
Link bandwidth $B_{e}$ (Gbit/s)	50	10∼100 (e∈E)
Computation capacity $D_{v}$ (Giga cycles/s)	30	10∼60 (v∈V)
Storage capacity $S_{v}$ (GByte)	20	10∼40 (v∈V)
Traffic rate $\lambda^{k}$ (Gbit/s)	25	10∼50 (k∈K)

## 2 Experimental Design, Materials and Methods

### 2.1 Network topologies

We describe hereafter the process we followed to generate the proposed network topologies, starting from *random* ones and then moving to a *real network scenario* where we used the localization information of several hundreds of Base Stations of a real mobile operator in the Milan city center.

We would like to point out that the main issue for the scientific community involved in testing optimization models and algorithms on real mobile network topologies is that it is not so easy to have access to the true topology interconnecting Base Stations (BSs), since such information concerning the network architecture is both quite sensitive for the mobile operator and also in continuous evolution. For this reason, it is unlikely that even just the Radio Access Network (RAN) topology of a mobile operator is known and publicly available, since it would provide sensitive details on its mobile network. At the same time, also the information about where edge servers are installed in a MEC architecture is generally not publicly available; typically, it depends on the chosen architecture and on the providers that the mobile operator decides to use. Some operators do not necessarily install one server in each cell. In fact, according to the specifications, the Edge server is an entity that can be installed in a completely separate network with respect to the access network or it can be installed in the same cabinet of the BS. Hence, knowing the cell location does not necessarily provide the servers’ topology.

For all these reasons, we decided to infer and make publicly available the ones described below.

#### 2.1.1 Random graphs

Erdös-Rényi random graphs [4] with different numbers of nodes (from 25 to 100) and edges (from 50 to 150) are generated. Note that, following such procedure, isolated nodes and components may exist in the generated graphs. To generate a connected network graph, for each isolated node v we first randomly sample nodes from the graph (the number of sampled nodes is chosen equal to the average node degree), and then connect them to v. Finally, we randomly eliminate *extra* edges to meet the desired number of edges while verifying that we still produce a connected graph. These topologies (indicated as 25N50E, 50N50E and 100N150E) can be viewed as representatives of Edge Network configurations where multiple edge nodes are scattered in different ways over a certain territory.

#### 2.1.2 A real network scenario

We further considered a real network scenario, with the actual deployment of Base Stations (BSs) collected from the open database OpenCellID [2]. Specifically, we considered the “Milan City Center” area around *Duomo* and selected one mobile operator (Vodafone) with 234 LTE (Long Term Evolution) cells falling in such area (see Fig. 2a). The BSs deployment shows where the BSs are located, while it does not show their interconnection topology nor where the edge clouds are deployed, since, as discussed before, such piece of information is both sensitive for the mobile operator and in continuous evolution.

Therefore, from the BSs location we generate a possible topology for the selected “Milan City Center” area through the following steps.

• We first perform a clustering on the LTE cells and assume MEC nodes are deployed only at the center of the clusters. This is to take into account that deploying the same number of MEC nodes as BSs would result in high expenditures by the network operators. The number of clusters could depend on the deployment budget of the network operator. Fig. 2b shows the details of the clustering, where, using the *KMeans* [5] algorithm, we obtain 30 clusters marked with different colors (corresponding to the number of cells belonging to each cluster). Note that other clustering methods can also be employed, like *DBSCAN* [6], a density based clustering algorithm, which does not require to specify the number of clusters but requires density parameters, e.g., the maximum distance between two nodes and the minimum number of nodes in a neighborhood to be considered. *KMeans* is more suitable in our case for directly controlling the number of clusters and, as a consequence, the network expenditure.
• Then, we generate a geometric graph by connecting any two nodes (cluster centroids) if the distance is lower than a given threshold (we fixed such parameter to 800 m). In this way, a dense graph with many links among the MEC nodes is produced, which might not be cost-effective due to the high costs for setting up links.
• Finally, we generate the Minimum Spanning Tree of the geometric graph weighted by the link distance and cluster size, which can represent a least total costs solution for setting up the network links. Moreover, to increase the redundancy and hence the reliability of the topology, we further preserve more links that connect some *small* nodes to the corresponding *aggregation node*, i.e., a node that is reached by multiple other nodes, as it happens in real networks. The final topology is illustrated in Fig. 2c, and edge clouds can be installed in all nodes (as suggested by 5G specifications).

### 2.2 Network parameters - motivation

The parameter settings proposed in this paper (see Table 2) are in line with those proposed in the various recent works. Among others, we cite the following: the white paper [7] illustrates the 5G testbed deployed by Cosmote (a Greek network operator, also provider of an Edge Computing infrastructure), which is based on an Openstack-based multi-cloud infrastructure interconnected with 10 Gbps fiber/copper links. The survey in [8] illustrates network capabilities and requirements of MEC hosts in smart metropolitan areas, and shows that every MEC host can use, on average, at least 62.5 Gbps in downlink and 10.41 Gbps in uplink. In [9], the authors study the network requirements to implement a use case of MEC-based Augmented Reality assisted remote surgery, which requires a bandwidth of at least 30 Gbps.

## Ethics Statement

Not applicable.

## CRediT authorship contribution statement

**Bin Xiang:** Conceptualization, Methodology, Software, Writing – original draft. **Jocelyne Elias:** Conceptualization, Writing – review & editing. **Fabio Martignon:** Conceptualization, Writing – review & editing. **Elisabetta Di Nitto:** Conceptualization, Writing – review & editing.

## Declaration of Competing Interest

The authors declare that they have no known competing financial interests or personal relationships that could have appeared to influence the work reported in this paper.
